# Association of *GST* Genes Polymorphisms with Asthma in Tunisian Children

**DOI:** 10.1155/2007/19564

**Published:** 2007-03-19

**Authors:** Chelbi Hanene, Lachheb Jihene, Ammar Jamel, Hamzaoui Kamel, Hamzaoui Agnès

**Affiliations:** ^1^Homeostasis and Cell Dysfunction Unit Research 99/UR/08-40, Faculty of Medicine, University of Tunis El Manar II, Tunis 1007, Tunisia; ^2^Department of Respiratory and Pediatric Diseases, Pneumology Hospital A. Mami, Ariana 2080, Tunisia

## Abstract

*Background*. A positive association between genetic polymorphism and asthma may not be extrapolated from one ethnic group to another based on intra- and interethnic allelic and genotype frequencies differences. *Objective*. We assessed whether polymorphisms of *GST* genes (*GSTM1*, *GSTT1,* and *GSTP1*) are associated with asthma and atopy among Tunisian children. *Methods*. 112 unrelated healthy individuals and 105 asthmatic (73 atopic and 32 nonatopic) children were studied. Genotyping the polymorphisms in the *GSTT1* and *GSTM1* genes was performed using the multiplex PCR. The GSTP1 ILe105Val polymorphism was determined using PCR-RFLP. *Results*. *GSTM1* null genotype was significantly associated with the increased risk of asthma (*P* = .002). Asthmatic children had a higher prevalence of the GSTP1Ile105 allele than the control group (43.8% and 33.5%, respectively; *P* = .002). Also, the presence of the GSTP1 homozygote Val/Val was less common in subjects with asthma than in control group. We have found that *GSTT1* null genotype (*GSTT1* *0/*0) was significantly associated with atopy (*P* = .008). *Conclusion*. Polymorphisms within genes of the *GST* superfamily were associated with risk of asthma and atopy in Tunisia.

## 1. INTRODUCTION

Asthma is a chronic disease characterized by reversible airflow obstruction and airway inflammation that affect many people. There is evidence that a genetic predisposition may also alter the capability of the airway to protect itself against inhaled toxic substances from the environment [1, 2].

Several candidate genes were implicated in the development of atopy and asthma. It has been reported that prevalence of these candidate genes can vary considerably by ethnicity [3, 4]. Furthermore, data from given studies suggest that a positive association between genetic polymorphism and asthma or atopy may not be extrapolated from one ethnic group to another based on intra- and interethnic allelic and genotype frequencies difference.

In North Africa, and especially in Tunisia, research data on this subject is absent. That is why we selected among asthmatic candidate genes three genes that have been known to manifest remarkable inter- and intraethnic differences [5]. These genes are *GSTT1*, *GSTM1*, and *GSTP1*, which are the code names for enzymes belonging to the glutathione S-transferase (*GST*) super family. In humans, *GSTs* represent a large and diverse super family of enzymes, with at least 13 *GST* enzymes belonging to five different families: mu, theta, alpha, pi, and gamma. The *GSTM1*, *GSTT1*, and *GSTP1* belong, respectively, to the GSTmu, GSTtheta, and GSTpi categories of enzymes. *GSTs* are known to play an important role in the functioning of antioxidant defences through reactive oxygen species (ROS) metabolism, in the repairing of damaged ROS and in the detoxification of several xenobiotics such as carcinogens found in tobacco smoking [6, 7]. The role played by *GSTs* may be especially important in response to oxidative stress [6, 8]. Common homozygote deletion polymorphisms of the *GSTM1* and *GSTT1* genes, as well as the GSTP1Ile105 polymorphism, have been known to abolish enzymes activity and increase susceptibility to oxidative stress [6, 8]. Studies have so far reported contradictory results regarding any association between *GST* gene polymorphisms and asthma and/or atopy. In fact, some have reported the presence of an association between GSTP1VAL105Ile polymorphisms and bronchial hyper responsiveness (BHR), asthma and atopy [9–16]. However, other studies have found no such evidence of any association between polymorphism and asthma or atopy [17]. In several studies, null alleles in the *GSTT1* and *GSTM1* genes (GSTT1*0 and GSTM1*0) were associated with childhood asthma [10, 11, 18, 19] but this finding was contradicted in other studies [17].

Although there is a high incidence of asthmatic diseases in Tunisia, no data has been reported in this country. In this study we assess whether polymorphisms of *GST* genes previously found to be associated with asthma and atopy in Caucasian and Asiatic subjects are also to be associated with asthma and atopy in Tunisian children.

## 2. SUBJECTS AND METHODS

### 2.1. Study subjects

Asthmatic children's histories were recorded using standard questionnaire categories: age, sex, exposure to tobacco smoke, and family history of asthma and allergy.

A total of 105 asthmatic children ranging from ages of 5 to 16 years (mean 11.5) were enrolled in this study along with 112 control individuals (aged 5 to 16 years, mean 9.5). None had any recent illnesses requiring treatment and no history of chronic diseases. All lived in the countryside near Tunis, in a town called Ariana. This region is generally considered to be representative of the general Tunisian population. Our national ethics committee approved the study.

### 2.2. Total IgE and Prick test assays

Atopy was defined by the skin sensitivity to specific allergens (skin reaction with a mean weal diameter ≥ 3 mm larger than that produced with one or more antigens in the presence of positive histamine control and a negative uncoated control) and by measurement of the total IgE level. Positive values were taken to be ≥ 200 UI/ml.

Among the 105 asthmatic children there were 73 atopic and 32 nonatopic children who had negative skin test responses to common allergens. In our study, asthma is frequently related to a heterogeneous group of clinical disorders including rhinitis, sinusitis, and dermatitis. The clinical profiles are shown in Table 1.

### 2.3. DNA isolation

The genomic DNA for genotyping was isolated from 10 ml of peripheral blood lymphocytes which were collected, using a salting-out DNA extraction procedure [19], in an EDTA containing a vacutainer.

### 2.4. GSTM1 and GSTT1 genotypes

The *GSTM1* and *GSTT1* null genotypes were detected using a multiplex PCR method [20]. Briefly, 100 ng of DNA were amplified in a-50 ul multiplex reaction mixture containing 0.90 pmol of each of the following GSTM1 primers (GSTM1-F: TTCCTCACTGGTCCTCACATCTC and GSTM1-R: TCACCGGATCATGGCCAGCA) and GSTT1 primers (GSTT1-F: GAACTCCCTGAAAAGCTAAAGC and GSTT1-R: GTTGGGCTCAAATATACGGTGG). As an internal control, the *ALBUMIN* gene was also amplified with 0.2 pmol of each primer (AlbF: GCCCTCTGCTAACAAGTCCTAC and AlbR: GCCCTAAAAAGAAAATCGCCAATC) in a medium consisting of 3.5 mM MgCl_2_, 200 uM dNTPs, 5 ul 10X PCR buffer, and 2U TaqDNA polymerase. The PCR protocol included an initial melting temperature of 94°C (5 minutes) followed by 35 cycles of amplification (20 seconds at 94°C, 20 seconds at 64°C, and 30 second at 72°C). A final 7-minute extension step (72°C) terminated the process.

The PCR products were analyzed on agarose gels. A fragment of 215 pb indicated the presence of GSTM1; a fragment of 480 pb indicated the presence of *GSTT1*; and a fragment of 380 pb indicated the positive internal control albumin. The subjects were classified as either (+), when at least one specimen of the gene was detected, or (−) when they showed a null genotype. Heterozygous individuals with *GSTM1* *(GSTM1+/− and GSTM1+/+)* or *GSTT1* *(GSTT1+/− and GSTT1+/+)* were reported to present similar enzymes activity [21] and expression levels [22] and were pooled together for statistical analysis.

#### 2.4.1. GSTP1 genotype

GSTP1 313A→G polymorphism (resulting in Ile105Val at codon 105) was analyzed by PCR-restriction fragment length polymorphism (RFLP) analysis [23]. Genomic DNA (100 ng) was used as a DNA template in a total 50 *μ*l volume reaction. The PCR products were digested in 25 *μ*l for 2 hours at 37°C with 5U Alw261. The digested products were then separated with 3% agarose gel stained with ethidium bromide. The presence of a-176 pb fragment indicated the wild-type genotype, whereas the 85 and 91 pb fragments indicated the homozygous polymorphic genotype. Heterozygous individuals recorded all three types of fragments.

## 3. STATISTICAL ANALYSIS

Association analysis in our case-control study was performed using standard Chi-squared test (Epistat statistical package, Epi Info Version 6) to detect differences in genotypes and alleles distribution among our groups.

Correction for multiple comparisons was performed, and only the value of corrected *P* < .05 was considered to be significant.

## 4. RESULTS

### 4.1. Case-control analysis

The association between *GST* genotype and susceptibility was studied in 105 unrelated asthmatic children residing in the northern part of Tunisia, using a control group of 112 healthy children.

Table 1 summarizes the clinical characteristics of subjects conducted in this study. In total, 35% of asthmatic children and 22% of nonasthmatic children were passive smokers. With an average age of 11.5 (ranging between 5 and 16 years), 70.32% of the asthmatic children were diagnosed as atopic.

Table 2 summarizes the data found regarding the genotype frequencies for the RFLP in the *GSTP1* gene, as well as the homozygous deletions of the *GSTM1* and *GSTT1* genes. Genotype frequencies (*GSTP1*, *GSTM1*, and *GSTT1*) were within the Hardy-Weinberg equilibrium for control population.

We found that *GSTM1* null genotype was significantly associated with increased risk of asthma (*P* = .002). Indeed, the *GSTM1* null genotype was present among 70.7% of the asthmatic children and among 50.2% of the control group.

As for the *GSTP1*, the homozygote *GSTP1* Val/Val genotype was less common among the asthmatic patients than in the control group (10.7% versus 18.6%, *P* = .04). Subjects with the *GSTP1Val/GSTP1Val* genotype registered a-2.33 fold lower risk of asthma than those with the *GSTP1Ile/Ile* genotype (OR = 2.33, 95% CL 0.94–5.87). Between both study samples, there was a significant difference in the frequency of the GSTP1 alleles (*P* = .02): asthmatic children had a higher prevalence of the GSTP1Ile allele than those in the control group (43.8% and 33.5%, resp.).

The presence of the *GSTT1* null polymorphism was compared in both sample groups. The difference showed to be nonsignificant (*P* > .05) between controls and asthmatics, 29.5% and 37.5%, respectively, see (Table 2).

### 4.2. GST genes and atopy

Table 3 summarizes the association between *GST* genes and atopy.

The *GSTT1* null genotype (GSTT1*0/*0) was significantly higher in atopic asthmatic cases than in nonatopic asthmatic subjects (*P* = .008). As for the *GSTM1*, there was a-1.5 fold increased risk of atopic asthma in individuals with the *GSTM1* null genotype (OR = 1.5; 95% CI, 0.6–3.83), but this increase was not significant. No significant associations have been found between atopy and GSTP1 polymorphism in present study.

### 4.3. Polymorphisms of glutathione S-transferase M1, T1, and P1 in a Tunisian control population

Human cytosolic *GSTs* have been well documented; they are polymorphic and have ethnic-dependent polymorphism frequencies. Compared with research carried out in other countries, the distribution of the *GSTM1* null genotype and the *GSTT1* null among our group of control was found to be, respectively, 50.2% and 29.5% (Table 4). Their GSTP1 polymorphism frequencies for the Ile/Ile genotype registered at 34.4%, at 47.1% for the Ile/Val genotype, and at 18.6% for the Val/Val genotype (Table 2). The Ile allele frequency for this particular group was set at 0.562.

## 5. DISCUSSION

The glutathione S-transferase (*GST*) super family of enzymes has a vital role in phase II of biotransformation of xenobiotics and in protection of cells from reactive oxygen species (ROS) by its ability to utilize substrates of a wide range of products of oxidative stress [6]. Oxidative stress was reported to be the key component of inflammation. Inflammation was considered a characteristic of asthma disease when it attacked airways. So defect in detoxifying ROS may influence the development and severity of asthma.

The results of our works suggest the presence of associations of *GSTM1*, T1, and P1 with childhood asthma and atopy. In comparing asthmatic children to healthy controls we have demonstrated a significant association between subjects lacking *GSTM1* activity and asthma (*P* = .002). Numerous studies have demonstrated a significant association between subjects lacking *GSTM1* activity and the risk of developing a form of lung disease [30–32]. For asthmatics, the association with *GSTM1* null genotype has been reported in Caucasian population [11, 18, 33, 34] but not in Asiatic groups [35].

As for the *GSTP1*, we have also found significant differences between our two study samples regarding the genotype frequencies of the GSTP1Ile105Val polymorphisms. Indeed, asthmatic children have low frequency of GSTP1Val allele compared with healthy children (*P* = .002). The defensive role of the *GSTP1* in cases of asthma was reported in several studies [9, 12–15]. It was reported that the presence of *GSTP1Val/Val* genotype conferred a sixfold lower risk of asthma than did GSTP1Ile/Ile and that the frequency of *GSTP1Val/Val* genotype correlated negatively with severity of airway dysfunction [9]. Aynacioglu et al. [12] have also reported that the frequency of GSTP1 Val homozygote was significantly lower in the group of patients with asthma than in the control individuals (3.8% *versus* 12.1%, *P* = .01). On the other hand, a recent study [11] has found that the GSTP1 Val/Val was more prevalent among asthmatic subjects than the control group (22.8% and 7.8%, respectively) and that subjects with the GSTP1 homozygous Val/Val genotype had a 3.55-fold increased risk of having atopic asthma compared to nonatopic asthma (OR = 3.55; 95% CI, 1.10–12.56), see [11]. Although, the *GSTP1*-derived enzyme contributes more than 90% of *GST* activity [36], it has been found by many studies [9, 12–15] and confirmed by our finding that it protects children against developing asthma disease. Nevertheless, it has been reported that the Val105 variant has higher catalytic efficiency for polycyclic aromatic hydrocarbon diol epoxides but its efficiency for 1-chloro-2, 4-dinitrobenzene is lower compared to the Ile105 variant [37]. Therefore, it seems to be possible that *GSTP1* plays a role in asthma disease by modulation of ROS production.

In this study, for *GSTT1* null genotype, significant association was found between atopic asthmatic children and nonatopic asthmatic children (*P* = .008), see (Table 2). While our findings are substantiated by several studies on Caucasians populations [11, 33], other studies were unable to establish this association [17, 18, 34, 38]. Several studies have suggested that individuals with the GSTT1*0/*0 * (GSTT1 null)* genotype may be more susceptible to genotoxic damage and lung diseases than individuals with the *GSTT1* gene [30, 39].

Contradictory results were found in regards to *GSTT1*, *GSTP1*, and *GSTM1* [11, 17, 34, 35, 39]; ethnicity is the most important reason for these differences. That is why we have taken into consideration inter- and intraethnic characteristics in analyzing our findings.

In control populations with intra- and interethnic differences, frequent genetic deletion polymorphisms of *GSTM1* (GSTM1*0/*0) and *GSTT1* (GSTT1*0/*0) have been reported [5]. The distribution of the *GSTM1* null genotype and *GSTT1* null of our healthy control sample was found to be 50.2% and 29.5%, respectively, see (Table 2).

The frequency of *GSTM1* null genotype in our healthy controls (50.2%) seems to be within the frequency range reported for the Caucasian populations (Table 4). In fact, the *GSTM1* deletion frequencies range, respectively, from 50.4% to 58.00%, 49% to 63%, and 20% to 33% in Caucasian, Asiatic, and African control groups [5].

Regarding *GSTT1* null type, the frequency of deletion genotype in our Tunisian control group is set at 29.5%. Like the Egyptian population, Tunisia has a slightly higher frequency than that registered by Caucasian Europeans (between 13% and 22.3%) and Africans (between 19% and 26%). The frequency of the deletion genotype in the Tunisian population is closer to that of the Caucasian Americans (10%–36%) and is considerably lower than that reported for the Asiatic populations (45% to 53%).

In conclusion, we have demonstrated that polymorphisms of *GST* genes previously found to be associated with asthma and atopy in Caucasian and Asiatic subjects are also associated with asthma and atopy in Tunisian children. Therefore, *GST* genotypes may be useful in order to optimize future treatment of asthma in the cases of patients with a risk profile.

## Figures and Tables

**Table 1:** The clinical characteristics.

		Cases *n* (%)	Controls *n* (%)

	Male	62 (59)	62 (55.3)
	Female	43 (41)	50 (44.7)
	Sex ratio (female/male)	0.69	0.80
	Mean age (standard deviation)	11.5 (5–16)	9.5 (5–16)
	FEV1 (% predicted)**	96.1 ± 14.2	ND
	FVC (% predicted)***	97.4 ± 14.6	ND
	Atopic	73 (70.32)	0
	Nonatopic	32 (29.68)	112 (100)
	Passive smoking	*n*(35)	*n*(22)

Phenotypes associated	Rhinitis	15 (14.28)	0
	Sinusitis	7 (6.66)	0
	Dermatitis	5 (4.76)	0
	RGO*	8 (7.61)	0
	Asthma, dermatitis, and rhinitis	7 (6.66)	0
	Asthma, sinusitis, and rhinitis	5 (4.76)	0

*RGO: gastro-esophageal reflux.

**FEV1: forced expiratory volume in 1 second.

***FVC: forced vital capacity; ND, not determined.

**Table 2:** Association of genotype profile between asthmatics and controls. NS: *P* value > .05.

Asthmatics Controls										

Gene	Chromosomal location	Polymorphisms	Genotypes	(*n* = 105)	(%)	(*n* = 112)	(%)	OR	95% CI	*P* value

*GSTM1*	1p13	Null allele	Null	79	70.7	53	50.2	2.35	1.30–4.27	.002
			WT	33	29.3	52	49.8			

*GSTT1*	22q11	Null allele	Null	42	37.5	31	29.5	1.43	0.78–2.63	NS
			WT	70	62.5	74	70.5			

*GSTP1*	11q13	Ile105Val	Ile/Ile	49	43.8	35	34.4	1.32	0.70–2.47	NS
			Ile/Val	51	45.5	48	47.1	1.77	0.73–4.35	NS
			Val/Val	12	10.7	20	18.6	2.33	0.94–5.87	.04
			A(Ile)	149	66.5	118	56.2	1.55	1.03–2.33	.027
			G(Val)	75	33.5	92	43.8			

**Table 3:** Association between genotype profile and atopic asthma. NS: *P* value > .05.

				Atopic asthmatics		Nonatopic asthmatics	

Gene	Chromosomal location	Polymorphisms	Genotypes	(*n* = 73)	%	(*n* = 32)	%	OR	95%CI	*P* value

*GSTM1*	1p13	Null allele	Null	37	50.7	13	40.6	—	—	NS
			WT	36	49.3	19	59.4	1.5	0.6–3.80	

*GSTT1*	22q11	Null allele	Null	36	49.31	7	21.8	—	—	.008
			WT	37	50.69	25	78.12	3.74	1.23–10.15	

*GSTP1*	11q13	Ile105Val	Ile/Ile	31	42.5	13	43.0	—	—	—
			Ile/Val	34	46.50	12	40.0	0.87	0.31–2.41	NS
			Val/Val	08	11.00	07	23.3	1.85	0.49–7.08	NS
			A(Ile)	95	65	38	59.4	—	—	—
			G(Val)	51	35	26	40.6	1.27	0.67–2.43	NS

**Table 4:** Comparison frequencies of *GSTM1* and *GSTT1* gene polymorphisms in Caucasian control populations.

Country	*GSTM1* null	*GSTT1* null	Reference

Tunisia	50.33% (105)*	29.50% (105)	Present study
Egypt	55.50% (200)	29.50% (300)	[24]
United Kingdom	57.80% (1122)	20.50% (922)	[5]
Sweden	55.90% (544)	13.00% (423)	[5]
France	53.40% (1184)	16.80% (512)	[5]
WhiteBrazilians	55.50% (233)	22.30% (233)	[25]
Canada	58.00% (90)	22.00% (90)	[26]
Spain	54.00% (200)	ND	[27]
Netherlands	50.40% (419)	22.90% (419)	[5]
Germany	53.00% (219)	20.00% (219)	[28]
Turkey	51.90% (133)	17.30% (133)	[29]

* Numbers between brackets represent the sample size; ND, not determined.

## ABBREVIATIONS

*GST:*: Glutathione-S-transferase
OR: Odds ratio
ROS: Reactive oxygen species
PCR-RFLP: Restriction length polymorphism
PCR: Polymerase chain reaction
